# Evaluation of PC12 Cells’ Proliferation, Adhesion and Migration with the Use of an Extracellular Matrix (CorMatrix) for Application in Neural Tissue Engineering

**DOI:** 10.3390/ma14143858

**Published:** 2021-07-10

**Authors:** Katarzyna Gębczak, Benita Wiatrak, Wojciech Fortuna

**Affiliations:** 1 Department of Basic Medical Sciences, Wroclaw Medical University, Borowska 211, 50-556 Wroclaw, Poland; benita.wiatrak@umed.wroc.pl; 2 Department of Pharmacology, Faculty of Medicine, Wroclaw Medical University, Mikulicza-Radeckiego 2, 50-345 Wroclaw, Poland; 3 Department of Neurosurgery, Wroclaw Medical University, Borowska 213, 50-556 Wroclaw, Poland; wojciech.fortuna@umed.wroc.pl

**Keywords:** CorMatrix, bioscaffold, PC12 cells

## Abstract

The use of extracellular matrix (ECM) biomaterials for soft tissue repair has proved extremely successful in animal models and in some clinical settings. The aim of the study was to investigate the effect of the commercially obtained CorMatrix bioscaffold on the viability, proliferation and migration of rat pheochromocytoma cell line PC12. PC12 cells were plated directly onto a CorMatrix flake or the well surface of a 12-well plate and cultured in RPMI-1640 medium and a medium supplemented with the nerve growth factor (NGF). The surface of the culture plates was modified with collagen type I (Col I). The number of PC12 cells was counted at four time points and then analysed for apoptosis using a staining kit containing annexin V conjugate with fluorescein and propidium iodide (PI). The effect of CorMatrix bioscaffold on the proliferation and migration of PC12 cells was tested by staining the cells with Hoechst 33258 solution for analysis using fluorescence microscopy. The research showed that the percentage of apoptotic and necrotic cells was low (less than 7%). CorMatrix stimulates the proliferation and possibly migration of PC12 cells that populate all levels of the three-dimensional architecture of the biomaterial. Further research on the mechanical and biochemical capabilities of CorMatrix offers prospects for the use of this material in neuro-regenerative applications.

## 1. Introduction

The fundamental part of tissue stroma is the extracellular matrix (ECM). ECM is the release product of cells settling on a given tissue; that is why both the composition and distribution of ECM particles depend on the tissue that ECM is derived from [1,2]. ECM maintains the particular tissue phenotype; influences the functioning of numerous regulatory growth factors and the availability of chemokines; and also has a significant impact on cell survivability, proliferation and cell differentiation [3]. Inside ECM, there are ligands stimulating integrins and other cell surface receptors, thus influencing the intercellular signalling pathways [1]. The history of using native ECM proteins in the construction of biomaterials starts with coating biomaterial surfaces with purified proteins, e.g., medicinal product Biobrane^®^, which was a synthetic network bonded with pig collagen [4]. In the 1980s, dual-layer artificial skin Integra^®^ was made. It contained purified ECM components derived from cows and sharks [5]. By contrast, AlloDerm^®^, made of dead donor cells, became the first acellular dermal substitute used in the treatment of burns and breast reconstruction [6]. ECM proteins were also tested with regard to the possibility of using them in wound dressings and as cell carriers. Graftskin, approved for use in 2001, was the first human skin equivalent. In the product, bovine type-I collagen was used to support live fibroblasts for the purpose of facilitating the healing process [7]. Recently, special acellular materials have been used in the same process; they contain growth factors derived from the urinary bladder and placental bed [8,9,10]. They constitute a non-toxic in vitro environment and promote adhesion, infiltration and proliferation of human fibroblasts and keratinocites. According to the latest research, ECM may also promote the disease process by providing physicochemical signals in the fibrosis process [11]. The source of ECM proteins may be various organs and tissues whose proteins are obtained by mechanical destruction, enzymatic digestion, chromatography and precipitation. By contrast, bacterial and eucaryotic cell lines are used to produce recombinant ECM proteins, such as fragments of human recombinant fibronectin [12,13,14], various types of collagen [15,16], human tropoelastin [17,18] and laminin [19,20]. Bioscaffolds obtained as a result of decellularization are more desired than modified ECM synthetic constructions, as they ensure outcomes which are more optimal and closer to the natural environment for cell growth and functioning [21].

Collagen, being a biomaterial of animal origin, is widely used as a basic component of the extracellular matrix and it has impact on the structural composition of cell nuclei as well as cell and tissue phenotype [22]. It is one of the major components of skin, tendons and cornea, and it also builds the bone and teeth environment by participating in in vitro regeneration and remodelling [23]. There are 28 collagen types differing in location, structure and function. Type I collagen forms a ligand for integrin β1 subunit [24] and glycoprotein VI [25], stimulating cells in their proliferation, migration and adhesion, and improving angiogenesis, which is essential in wound curing and tissue repair processes. It can also self-organize in particular physiological conditions, and electrospinning of collagen nano-fibres allows them to form spheroids influencing the survivability and differentiation of human bone marrow-derived mesenchymal stem cells (BM-MSC) in the orientation of cardiomyocytes [26]. Owing to the fact that it is a carrier in the encapsulation of stem cells, it is used in skin regeneration processes [27]. The model of the collagen–GAG scaffold allows for the control of the differentiation of mesenchymal stem cells by modulating the growth factor release, which may be applied in muscle-skeleton regenerative medicine [28]. Recent research has indicated that collagens participate in axon growth, synaptogenesis and Schwann cell differentiation [29].

Biological scaffold materials are obtained from various tissues, including heart valves [30,31], blood vessels [32,33], skin [34], nerves [35], skeletal muscles [36], tendons [37], ligaments [38], small intestinal submucosa SIS [39,40,41], urinary bladder [42], and liver [43]. The largest amount of comparative research on the mechanical and structural potential of ECM scaffolds was conducted using urinary bladder material (UBM) and pig small intestinal submucosa (SIS). The best characterized material was SIS; it contained 90% collagen, mostly type I, and various gligozaminoglicans, including heparin, heparan sulphate, chondroitin sulphate and hyaluronic acid [2]. It was discovered that ionic detergents may lead to the loss of GAG through ECM [44]. SIS-ECM also contains adhesive particles, such as fibronectin or laminin, decorin [45], glycoprotein biglycan, entactin and various growth factors: TGF-β, b-FGF and VEGF [2]. The presence of growth factors increases fibroblast migration and matric integration in the recovery processes of the heart and ligaments [46,47].

SIS was used for the first time in 1989 as an autograft of a large vessel in a dog; however, earlier in the 60 s, it was used also in experiments on vessel grafts. SIS-ECM was used in clinical applications in over 1 m patients. After the capsulation of gingival mesenchymal stem cells, SIS-ECM induced the regeneration of tongue mucosa and the mucosa barrier in ulcerative colitis [48,49]. There are a number of pig-derived SIS products (e.g., Surgisis^®^, Durasis^®^, Stratasis^®^; Cook Biotech, Lafayette, IN, USA; Restore^®^; DuPuy, West Chester, PA, USA) [50]. One of the commercially obtained products is CorMatrix, produced by the decellularization of pig small intestinal submucosa. It is characterized by three-dimensional architecture allowing the ingrowth of host cells; it also initiates and controls cell proliferation and differentiation, and is characterized by low immunogenicity. CorMatrix was granted the Food and Drug Administration and CE certificates for medical products used as patches in pericardium reconstruction and repair, and heart and carotid artery repair. There are similar, commercial ECM products used in heart and vessel damage (e.g., MatrixP^®^, Autotissue, Berlin, Germany, and CardioCel^®^, Admedus, Perth, Australia) [50]. CorMatrix has recently been successfully used in ischemic heart disease in an ox; the proliferation and perfusion values were the highest in comparison to other ECM [51,52]. CorMatrix has been used in cardiovascular surgery since 2010, among others, in the surgical treatment of congenital heart and vascular defects [53,54], pericardium reconstruction [55,56,57,58], valve reconstruction in adults and children [55,57,59], endocardium reconstruction [60], and the repair of post-myocardial infarction defects [61].

There is research indicating that ECM has impact on the proliferation, migration and differentiation of rat-derived neural and precursor stem cells, the proliferation of mouse-derived neuroepithelial cells [62], and the migration of mouse cerebellum precursor cells derived in vitro [63].

Line PC12 is derived from pheochromocytama; geoplastic rat cells were obtained from neural crest tissues [1]. There are many kinds of PC12 cells described in scientific reports. The suspension type of PC12 line grow as small and irregularly shaped, have some features of neurons and under the influence of nerve growth factor (NGF) resemble the neurons of the cortex; its use is recommended in studies of the nervous system. It is used as a model of neuronal cells in in vitro tests on mitogenesis, chemotaxis and differentiation. This cell line has been used previously to study mechanisms of action of various neurotoxic substances. These cells are widely used as a model for neurodegenerative diseases, such as Alzheimer’s disease and Parkinson’s disease.

The subject of the present study was the investigation of the behavior of line PC12 cells in in vitro cultures using various substrates: collagen, non-ECM polymer (poly-D-lysine; PDL) and commercial biomaterial CorMatrix, as well as the assessment of the possible use of CorMatrix as a biological scaffold in neuro-regenerative medicine.

## 2. Materials and Methods

### 2.1. Material

CorMatrix (CorMatrix Cardiovascular, Roswell, GA, USA) is a commercially available material created by the decellularization of the small intestinal submucosa of pigs. It has a three-dimensional structure that allows ingrowth of host cells, initiates and maintains control over cell proliferation and differentiation and has low immunogenicity. CorMatrix is certified by the Food and Drug Administration as a medical product and has been used in cardiovascular clinical surgery since 2010, including surgical treatment of congenital heart and vascular diseases, pericardium reconstruction, valve reconstruction in adult and child endocarditis, and repair of damage after myocardial infarction.

### 2.2. Cell Line

Assessment of sterile CorMatrix^®^ECM^®^ material properties was carried out on the PC12 cells obtained from Sigma-Aldrich (St. Louis, MO, USA). This line is derived from the rat adrenal pheochromocytoma tumor. In the ATCC collection are distinguished two types of this cell line—suspension and adherent. The adherent line was derived from the suspension line as a result of multiple passages. A suspension line was chosen for further investigation. The study was carried out using cells between 15 and 25 passages because at higher passages, there is too much morphological variability. After treatment with nerve growth factor (NGF), these cells become similar to sympathetic neurons, and it has been recognized that they can be a model in neurobiological research. The cells were passaged twice a week.

### 2.3. Media and Conditions

Cells were cultured and seeded on multi-well plates for experiments in RPMI-1640 medium supplemented with 10% DHS, 5% FBS, 2 mM L-glutamine and 25 μg/mL gentamicin (complete medium, Lonza, Basel, Switzerland). After 24 h of cell regeneration, the medium was replaced with RPMI-1640 supplemented analogously with antibiotics, but with serum content reduced to 1% DHS, and with the addition of 100 μg/mL NGF. All of the studies were carried out in 5% CO_2_, 37 °C and 95% humidity.

### 2.4. Experimental Model

**Scheme 1:**
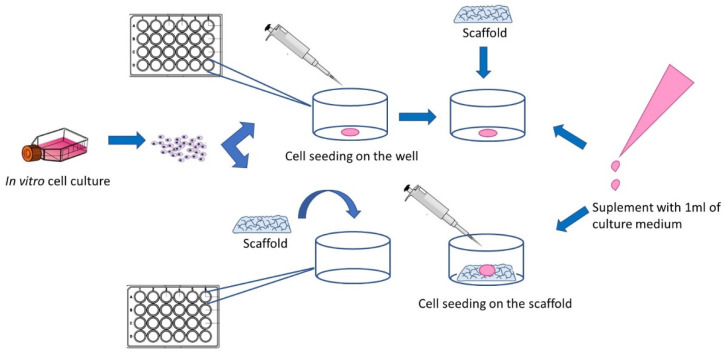
The illustration explains the procedure of cell culture with CorMatrix in the two ways.

Two types of 12-well plates were used in experiments—plates provided from the producer (Merck KGaA, Darmstadt, Germany) and plates with the modified surface of the culture wells with collagen. The modification was carried out using collagen type I solution at a concentration of 0.01% (*w*/*v*) overnight at 4–8 °C. Before use, the plates were exposed to UV light for 30 min for sterilization. CorMatrix material was cut into 10 mm × 10 mm fragments and then used in experiments in two ways. PC12 cells were seeded at a density of 50,000 cells/well directly onto the material, or the material without cells was put into a well with previously seeded cells. For the adherence of cells to the CorMatrix material, 50,000 cells were seeded directly onto it in a volume of 100 μL medium. After 30 min of incubation, the solution in the well was diluted to a final volume of 1 mL as illustrated in Scheme 1. Two types of control were used in the experiments. The first control cells were cultured only in a complete medium, the second in a medium containing NGF. Observations and evaluations were carried out at 4 time points: one day after seeding and after 3, 6 and 9 days of incubation with NGF. The number of cells in the collected medium, adhered to the CorMatrix material and adhered to the surface of wells were counted. The PC12 cells were counted on the three different levels of the CorMatrix material. The study involved 4 independent experiments under repetitive conditions, each of which included 3 replicates.

### 2.5. Detection of Apoptosis

Cell apoptosis was checked using a staining kit provided by Sigma-Aldrich (Sant Luis, MO, USA), containing annexin V conjugated with fluorescein and propidium iodide. The PC12 cells were transferred to test tubes and incubated with annexin V and PI solution in the dark at RT for 20 min. The viability of the culture was assessed by the image-based cytometer Arthur (living, apoptotic and necrotic cells).

### 2.6. Analysis of the Number of Cells on the CorMatrix Material

In order to count cells adhered to the CorMatrix material and to the surface of the wells, the PC12 cells were stained with Hoechst 33258 solution for 1 h in the dark at 37 °C. Then, using fluorescence microscopy, 5 photos were taken in various areas of the surfaces of the well and CorMatrix material (on the three different levels). The mean number of cells in the field of view was analyzed. To determine the number of cells in the image, our own software using the OpenCV library was used. Cell counting software implemented, among others, the watershed segmentation algorithm; this proved to be the most effective in the separation of cells. All photos were analyzed with the same application settings, which allowed us to reduce random errors of interpretation.

### 2.7. Statistical Analysis

The results were analyzed according to generally accepted principles. Initially, the normal distribution was checked using the Shapiro–Wilk test and equality of variance using Levene’s test. Kruskal–Wallis test and appropriate post-hoc analysis were performed.

## 3. Results

The performed cell apoptosis test showed that the number of apoptotic and necrotic cells did not exceed 7% in total for all tested cases (data not shown).

### 3.1. Evaluation of the Effect of CorMatrix Material on PC12 Cells Proliferation

The number of cells in the medium and CorMatrix material and the surface of the wells modified with type I collagen and without modification were calculated. Assessments were made at four time points: on the 1st, 3rd, 6th and 9th day of cultivation.

**Figure 1:**
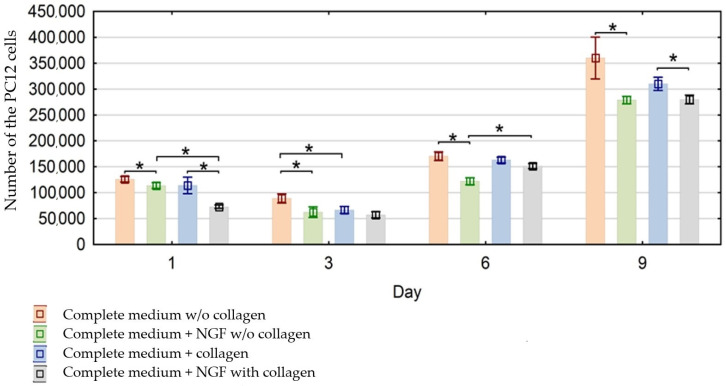
Number of cells in the collected medium (cell cultures without CorMatrix material); * *p* < 0.05—significant difference in number of cells.

The number of cells in the collected complete medium and medium containing NGF is shown in Figure 1, taking into account the influence of modified and unmodified plates on the culture. A greater increase in proliferation of cells growing in suspension was observed on unmodified plates. On the other hand, modification with collagen reduced cell division due to increasing the adherence of cells to the surface of the wells.

Incubation with nerve growth factor (NGF) inhibited the proliferation of PC12 cells in the following days of culture (on day 9, the reduction in proliferation was statistically significant compared to day 6, regardless of the type of well surface). In the presence of NGF, the proliferation phase is stopped. Earlier surface modification with type I collagen allowed the cells to adhere to the surface, causing PC12 cells to differentiate faster than cells normally grown in suspension (Figure 1).

**Figure 2:**
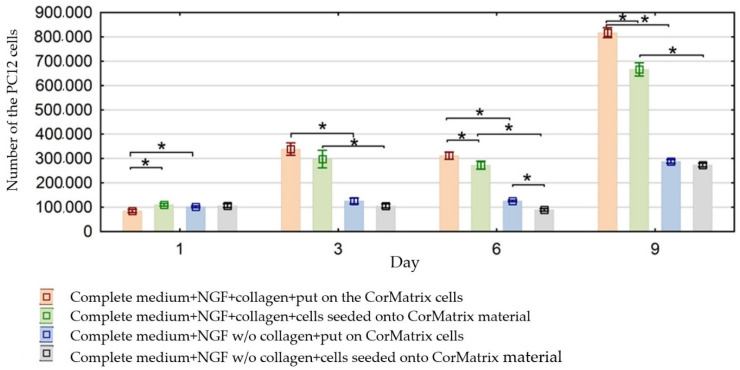
Number of cells in the collected medium (cell cultures with CorMatrix material); * *p* < 0.05—significant difference in number of cells.

Cell cultures with CorMatrix material were sown in two ways: cells were either sown directly on the material or the material was placed on the surface of the medium with cells. In these two cases, the number of cells in the harvested medium was also assessed. The number of cells in the following days of culture (1, 6 and 9) was significantly lower (*p* < 0.05) when the cells were plated directly onto the material (Figure 2).

Regardless of the method of sowing cells in cultures with CorMatrix material and collagen-modified dock surface, cell proliferation in the following days of culture (3, 6 and 9) was greater than in unmodified plates (Figure 2). The greatest increase in the number of cells between consecutive days of the experiment was observed in type I collagen-modified wells and when the CorMatrix material was placed on the surface of the medium with cells.

### 3.2. Evaluation of the Effect of CorMatrix Material on PC12 Cells Migration

**Figure 3:**
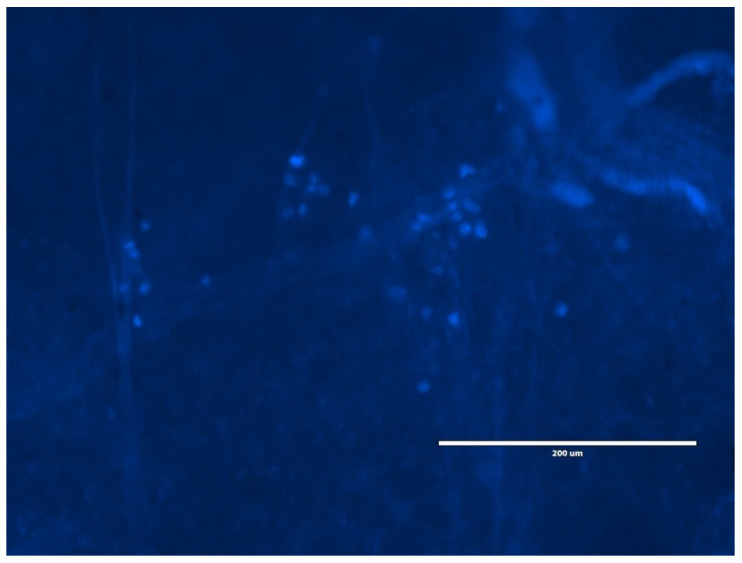
Sample microphotograph of Hoechst 33258 stained PC12 cells on the surface of the CorMatrix; scale bar: 200 µm.

Hoechst 33258-stained PC12 cells were counted using the ImageJ open-access platform. PC12 cells were grown in suspension on unmodified collagen plates. The mean number of cells in the field of view was in the range of 14–69 cells. In contrast, in cultures modified with type I collagen, cells adhered and differentiated in NGF’s presence. As for the cells on the surface of the CorMatrix, it could be seen that the cells adhered to the surface of the material (Figure 3).

Greater migration of PC12 cells to the surface of CorMatrix was observed than to the surfaces of both unmodified and collagen-modified wells. In the case of sowing cells into collagen-modified wells and putting them onto CorMatrix material cells, a much larger number of cells was counted on the surface of CorMatrix than on the well surface: on day 9, 1900–2900 cells were counted on the CorMatrix-2200, while on the collagen-modified surface only 300–500 cells were counted. However, the mean number of PC12 cells on the collagen-modified surface but without CorMatrix was 800–1500 in the field of view. This study suggests possible greater cell migration to CorMatrix than to the surface modified with type I collagen.

### 3.3. Evaluation of the Cell Number on the CorMatrix Material

**Figure 4:**
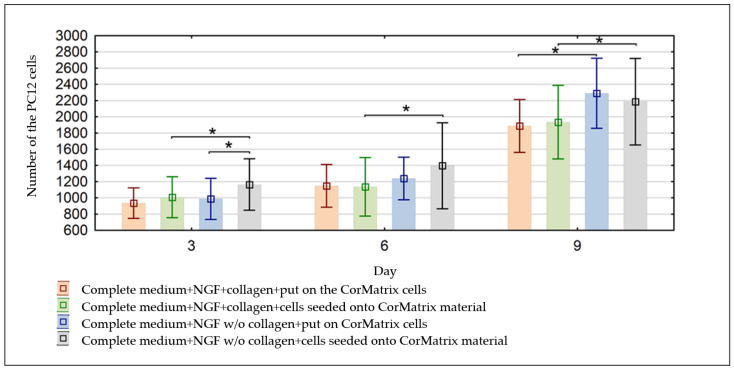
The average number of cells in the field of vision on the microphotographs of CorMatrix material; * *p* < 0.05—significant difference in number of cells.

Regardless of cell application and the type of surface of the culture plates, a statistically significant (*p* < 0.05) increase in the number of cells on the CorMatrix material was observed between all consecutive days of the study (Figure 4).

The effect of covering the surface of the wells on the number of cells was also assessed. The number of cells plated directly onto the CorMatrix (on days 3, 6 and 9) was statistically significantly (*p* < 0.05) greater when the well surfaces were not modified. In the case of the second method of CorMatrix application, the increase in the number of cells was higher on the day of culture also on unmodified plates.

The application of the material was also analyzed. On day 3 cultivation, the number of cells was statistically significantly (*p* < 0.05) higher when the cells were plated directly onto the material on unmodified plates. However, already by the 6th and 9th days, no differences were observed between the number of cells and the method of material application. This probably indicates the migration of cells to the surface of the tested material. At the same time, the lower number of cells on the 9th day on plates placed directly onto the material compared to placing the material on the surface of the medium with cells may suggest cell differentiation. On plates modified with type I collagen, no statistically significant differences in the number of PC12 cells were observed depending on the method of CorMatrix application.

## 4. Discussion

Biological scaffolds constitute a significantly better alternative to the artificial materials used in medical applications. Both ECM-derived scaffold material and artificial bioscaffolds have been used in multiple clinical applications with various degrees of success. For example, the use of the biologic scaffold in contaminated fields has allowed for one-stage Ventral hernia repair. ECM-derived biomaterial is a real and effective alternative for radical esophagectomy in esophageal repair [64]. Acellular dermal matrix (ADM) can be used in breast reconstruction [65], and in skin curing processes as it facilitates fibroblast and endothelial cell infiltration near an implant [66]. Adipose tissue can also be an ECM source. It has been shown that decellularized adipose tissue can stimulate the infiltration of adipogenous stem cells (ASC) together with adipose tissue macrophages, and also their differentiation [67,68]. Commercial CorMatrix is the most extensively used SIS-ECM product in cardiovascular surgery procedures, and its clinical applications as biological scaffolds are mostly related to intra- and extracardiac sites [50]. It results mainly from CorMatrix properties, such as reabsorption due to enzymatic in vivo degradation, the significant initial species/tissue-based elasticity, the porcine SIS [69]. The high extensibility of CorMatrix can be explained by strong interactions between longitudinal collagen fibres [70]. The use of SIS-ECM scaffolds in the reconstruction of the central nerve system (CNS) still remains at an early development stage. An example of central nerve system restoration is the use of a polymeric nano-fibrous scaffold, such as a biodegradable poly(L-lactic acid) (PLLA), for in vitro culture of nerve stem cells (NSCs) [71].

The purpose of this research was to test whether the acellular biologic scaffold, such as CorMatrix, is able to modulate the behavior of model neural-like cell PC12, whether it influences the increase in proliferation and the PC12 cell line in vitro migration, and as a result finding out if CorMatrix can be used in neural applications.

The in vitro research indicated a low percentage of apoptotic cells in the collected medium during the whole experimentation time (nine days). This confirms the high survivability of PC12 cell cultures in the presence of various matrices, such as type-I collagen and CorMatrix. The behavior analysis of bone marrow-derived mesenchymal stem cells isolated from adult male L2G85 transgenic FVB mice indicates long-term survivability of Matrigel-supported stem cells, even up to five months [72]. The in vivo research conducted by Uemura et al. in 2010 showed an increase in the number of cell deaths detected using TUNEL staining at 24 h in the graft with growth factor-reduced Matrigel (grfMG) and Ki-67 positive at 72 h. The initial decrease in cell survivability can be explained by the occurrence of stress and/or hypoxic conditions or the inhibition of the influx of macrophages which phagocitize cells in an early apoptotic phase [73].

In the conducted tests, it was observed that the number of cells in the collected medium on surfaces not modified with collagen increased and the number of cells in the medium containing NGF, regardless of the type of flask surface, decreased (PC12 cell culture without CorMatrix) (Figure 1). The decreased number of the PC12 cells on plates modified with collagen results from cell adherence to the surface, because collagen type I is an adhesive substrate for many types of neurons [29]. Simultaneously, cell culturing from NGF stops proliferation and starts the differentiation process. Even early research conducted on PC12 cells by Green and Tischler in 1976 showed that in response to NGF, PC12 cells differentiate into sympathetic neurons and elongate neurites [74]. On day 1, a significant increase in the number of cells in the collected medium enriched with NGF on plates not modified with collagen was observed in comparison with the surface modified with collagen; the opposite dependence was observed on day 6. The remaining days did not bring significant differences in either of the cases. This can be explained by the investigations conducted by Bachmann et al., who demonstrated that the pre-incubation of collagen type-I with the extracellular domain of myelin-associated glycoprotein (s-MAG) reduced the adhesion of PC12 cells by modulating the adhesion properties of collagens, in particular collagen type I, probably by modifying the integrin binding site [75].

In the case of PC12 cell cultures with CorMatrix, it was observed that the number of cells in the collected medium with NGF on days 3, 6 and 9 grew significantly on the collagen-coated surface in comparison with the unmodified one (Figure 2). On days 1, 6 and 9, the differences resulting from the method used to apply PC12 cells on the biological scaffold were determined. The number of cells in the collected material was smaller if they were applied on a patch. The application of PC12 cells on the tested biomaterial may facilitate their ingrowth in the CorMatrix structure and hence exclude accidental clustering in the collected medium. The commercially obtained CorMatrix is characterized by three-dimensional architecture allowing for the ingrowth of host cells; it initiates and maintains control over the proliferation and differentiation of cells and is characterized by low immunogenicity [50]. The research conducted by Herbikova et al. shows also that mouse myoblasts C2C12 adhered and penetrated into the decellularized skeletal muscle scaffold, and migrated through the extracellular matrix to be organized in a three-dimensional space. The presence of neuronal progenitor cells was also detected on the first day after the implantation of the ECM hydrogel in damaged rat brains; they differentiated into mature neurons and astrocytes in degrading bioscaffolds [76].

In the conducted research, a significant increase in the number of PC12 cells was observed at three different levels of the structure of used biomaterial on culture surfaces not modified with collagen (on days 3, 6 and 9) (Figure 4). CorMatrix was successfully introduced in a bovine model of chronic ischemia heart failure, where the highest level of cell proliferation and end-organ perfusion was observed in comparison with other methods of introducing ECM [51,52]. The experiments showed the influence of CorMatrix on both the proliferation and possible migration of the tested cell line, which demonstrates the increase in the number of cells in the tested biomaterial throughout the whole observation period (Figure 4) and also in the collected medium on day 9 of the experiment in the culture with a collagen modified surface (Figure 2). This may indicate the synergic effect of not only CorMatrix but also collagen on cell proliferation (Figure 2). The knitted silk scaffold combined with collagen reinforces the regeneration ability, offers better mechanical properties and creates a microstructure characterized by better organisation in comparison with the pure silk group. It was demonstrated in the long-term enhancement of the repair process in the rabbit anterior cruciate ligament (ACL) defect model by a clear growth of fibroblast-like cell proliferation [77]. It was shown that the biomaterial composed of collagen and laminin, reinforced with chitosan, fosters the survivability of circulating angiogenic cells. Collagen has also been used with synthetic polymers, such as polycaprolactone (PCL) and bioactive glass nanoparticles, with which it made a nanofibrous scaffold used as a carrier for endothelial progenitor cells and significantly accelerated the neovascularisation process in skin injury healing [18]. In other research, higher collagen content in collagen–alginate composite material increased the elasticity and osteogenic differentiation MSC [78]. In grafting after myocardial infarction, patches made of basement membrane (Basement Membrane Matrix) and poly(lactic-co-glycolic acid)/poly (L-lactic acid) polymers, enriched with angiogenic and pro-survival factors, and embryonal cardiomyocytes, endothelial cells and fibroblasts were used [79].

The obtained results indicate also the possible migration of cells to the CorMatrix material; PC12 cells tended to select the surface of the tested biomaterial rather than the collagen modified surface. ECM molecules exerted a significant influence on cell migration, which was shown in the research on the expression of cell adhesion molecules and extracellular matrix molecules in human brain development, tests on the influence of ECM components on rat oligodendrocyte progenitor cells (OPCs), and the investigations on the role of ECM in the regulation of rodent and human neural stem cell migration [3,80,81]. In the conducted experiments, no significant differences in the proliferation of PC12 cells depending on the method of application of a CorMatrix patch on the medium with cells or the cells on the patch were found. Only on day 3 of observation was a clear dependence on the method of application on the surface not modified with collagen reported (Figure 4). The increase in the number of PC12 cells in the case when the cells were applied on the material can be explained by the influence of CorMatrix on the proliferation and possible migration of an additional pool of cells to the patch. No differences on day 6 and 9 of the experiment can be explained by the cell migration inhibition mechanism resulting from the characteristic weave and mechanical properties of CorMatrix. As was demonstrated by Baldwin et al., the presence of fibronectin and laminin may influence the migration of cells through the collage matrix, due to a correlation with characteristic cross-linking where spaces between fibers are larger in the ECM gel, whose mechanical properties are modified in the gelation time. This was confirmed also by Kuntz and Salzman’s experiments (unpublished data), in which fibronectin and laminin inhibited the migration of human neutrophils through collage gels, where the size of PC12 cells is close to that of neutrophilia. PC12 cells have an effect of fibronectin and laminin present in ECM-derived materials owing to an integrin receptor, which may indicate the existence of another mechanism for the inhibition of cell migration. Comparative research on the central nervous system (CNS) ECM scaffold and non-CNS ECM scaffolds showed that the urinary bladder ECM (a non-CNS derived material) stimulated PC12 cells to proliferate; however, it simultaneously inhibited migration [1].

Limitations of the research carried out could include the subjective choice of the observation areas, as well as the lack of assessment of the level of cell culture differentiation towards neuronal cells.

## 5. Conclusions

Regenerative medicine encompassing a wide spectrum of diseases, ranging from cardio-vascular ones to neurodegenerative ones, constitutes an extensive area for ECM-derived biomaterial applications. Acellular biomaterials making biodegradable, three-dimensional scaffolds can be an alternative for cell transplants, whose most significant disadvantage is the low survivability of graft cells. Tissue-derived ECM scaffolds, owing to regulator growth factors and chemokines they contain, can shape factors of cell behavior, such as survivability, proliferation, migration and differentiation [82].

Cormatrix can constitute an important alternative to other biomaterials due to its innate abilities (1) to extend; (2) to integrate with host tissue; and (3) to create a micro-environment fostering the growth, possible migration and differentiation of cells. The application of CorMatrix material as a biological scaffold seems to be an interesting method in the repair of nerve tissue damage.

## Data Availability

The data presented in this study are available on request from the corresponding author.

## References

[1] Crapo P.M., Medberry C.J., Reing J., Tottey S., van der Merwe Y., Jones K., Badylak S.F. (2012). Biologic scaffolds composed of central nervous system extracellular matrix. Biomaterials.

[2] Badylak S.F., Freytes D.O., Gilbert T. (2015). Reprint of: Extracellular matrix as a biological scaffold material: Structure and function. Acta Biomater..

[3] Hu J., Deng L., Wang X., Xu X.-M. (2009). Effects of extracellular matrix molecules on the growth properties of oligodendrocyte progenitor cells in vitro. J. Neurosci. Res..

[4] Tavis M.J., Thornton J., Danet R., Bartlett R.H. (1978). Current Status of Skin Substitutes. Surg. Clin. N. Am..

[5] Burke J.F., Yannas I.V., Quinby W.C., Bondoc C.C., Jung W.K. (1981). Successful Use of a Physiologically Acceptable Artificial Skin in the Treatment of Extensive Burn Injury. Ann. Surg..

[6] Wainwright D. (1995). Use of an acellular allograft dermal matrix (AlloDerm) in the management of full-thickness burns. Burn.

[7] Falanga V.A., Armstrong D.G., Sabolinski M.L. (2001). Graftskin, a Human Skin Equivalent. Diabetes Care.

[8] Park T.H., Choi W.Y., Lee J.H., Lee W.J. (2017). Micronized Cross-Linked Human Acellular Dermal Matrices: An Effective Scaffold for Collagen Synthesis and Promising Material for Tissue Augmentation. Tissue Eng. Regen. Med..

[9] Meng L., Liao W., Yang S., Xiong Y., Song C., Liu L. (2016). Tissue-engineered tubular substitutions for urinary diversion in a rabbit model. Exp. Biol. Med..

[10] Rameshbabu A.P., Datta S., Bankoti K., Subramani E., Chaudhury K., Lalzawmliana V., Nandi S.K., Dhara S. (2018). Polycaprolactone nanofibers functionalized with placental derived extracellular matrix for stimulating wound healing activity. J. Mater. Chem. B.

[11] Klingberg F., Chau G., Walraven M., Boo S., Koehler A., Chow M.L., Hinz B. (2018). The ED-A Domain Enhances the Capacity of Fibronectin to Store Latent TGF-Beta; Binding Pro-tein-1 in the Fibroblast Matrix. J. Cell Sci..

[12] Amaral I., Neiva I., Silva F., Sousa S.R., Piloto A., Lopes C., Barbosa M., Kirkpatrick C., Pêgo A.P. (2013). Endothelialization of chitosan porous conduits via immobilization of a recombinant fibronectin fragment (rhFNIII7–10). Acta Biomater..

[13] Herranz-Diez C., Mas-Moruno C., Neubauer S., Kessler H., Gil F.J., Pegueroles M., Manero J.M., Guillem-Marti J. (2016). Tuning Mesenchymal Stem Cell Response onto Titanium–Niobium–Hafnium Alloy by Recombinant Fibronectin Fragments. ACS Appl. Mater. Interfaces.

[14] Yun Y.-R., Pham L.B.H., Yoo Y.-R., Lee S., Kim H.-W., Jang J.-H. (2015). Engineering of Self-Assembled Fibronectin Matrix Protein and Its Effects on Mesenchymal Stem Cells. Int. J. Mol. Sci..

[15] Wang Y., Zeinali-Davarani S., Zhang Y. (2014). Arterial mechanics considering the structural and mechanical contributions of ECM constituents. J. Biomech..

[16] Stoichevska V., Peng Y.Y., Vashi A.V., Werkmeister J.A., Dumsday G., Ramshaw J.A.M. (2017). Engineering specific chemical modification sites into a collagen-like protein fromStreptococcus pyogenes. J. Biomed. Mater. Res. Part A.

[17] Martin S.L., Vrhovski B., Weiss A.S. (1995). Total synthesis and expression in Escherichia coli of a gene encoding human tropoelastin. Gene.

[18] Wang L., Ding L., Yu Z., Zhang T., Ma S., Liu J. (2016). Intracellular ROS scavenging and antioxidant enzyme regulating capacities of corn gluten meal-derived antioxidant peptides in HepG2 cells. Food Res. Int..

[19] Kortesmaa J., Yurchenco P., Tryggvason K. (2000). Recombinant Laminin-8 (α4β1γ1). J. Biol. Chem..

[20] Rodin S., Domogatskaya A., Ström S., Hansson E.M., Chien K.R., Inzunza J., Hovatta O., Tryggvason K. (2010). Long-term self-renewal of human pluripotent stem cells on human recombinant laminin-511. Nat. Biotechnol..

[21] Xing H., Lee H., Luo L., Kyriakides T.R. (2020). Extracellular matrix-derived biomaterials in engineering cell function. Biotechnol. Adv..

[22] Lelievre S., Weaver V.M., Nickerson J.A., Larabell C.A., Bhaumik A., Petersen O.W., Bissell M.J. (1998). Tissue phenotype depends on reciprocal interactions between the extracellular matrix and the structural organization of the nucleus. Proc. Natl. Acad. Sci. USA.

[23] Uemura M., Refaat M.M., Shinoyama M., Hayashi H., Hashimoto N., Takahashi J. (2009). Matrigel supports survival and neuronal differentiation of grafted embryonic stem cell-derived neural precursor cells. J. Neurosci. Res..

[24] Hynes R.O. (1992). Integrins: Versatility, modulation, and signaling in cell adhesion. Cell.

[25] Smethurst P.A., Onley D.J., Jarvis G., O’Connor M.N., Knight C.G., Herr A., Ouwehand W.H., Farndale R.W. (2007). Structural basis for the platelet-collagen interaction: The smallest motif within collagen that recognizes and activates platelet Glycoprotein VI contains two glycine-proline-hydroxyproline triplets. J. Biol. Chem..

[26] Joshi J., Brennan D., Beachley V., Kothapalli C.R. (2018). Cardiomyogenic differentiation of human bone marrow-derived mesenchymal stem cell spheroids within electrospun collagen nanofiber mats. J. Biomed. Mater. Res. Part A.

[27] Altman A.M., Matthias N., Yan Y., Song Y.-H., Bai X., Chiu E.S., Slakey D.P., Alt E.U. (2008). Dermal matrix as a carrier for in vivo delivery of human adipose-derived stem cells. Biomaterials.

[28] Grier W.K., Tiffany A.S., Ramsey M.D., Harley B.A. (2018). Incorporating β-cyclodextrin into collagen scaffolds to sequester growth factors and modulate mesenchymal stem cell activity. Acta Biomater..

[29] Hubert T., Grimal S., Carroll P., Fichard-Carroll A. (2009). Collagens in the developing and diseased nervous system. Cell. Mol. Life Sci..

[30] Grauss R.W., Hazekamp M.G., Oppenhuizen F., Van Munsterena C.J., Groot A.C.G.-D., DeRuiter M.C. (2005). Histological evaluation of decellularised porcine aortic valves: Matrix changes due to different decellularisation methods. Eur. J. Cardio-Thoracic Surg..

[31] Schenke-Layland K., Vasilevski O., Opitz F., König K., Riemann I., Halbhuber K., Wahlers T., Stock U. (2003). Impact of decellularization of xenogeneic tissue on extracellular matrix integrity for tissue engineering of heart valves. J. Struct. Biol..

[32] Dahl S.L.M., Koh J., Prabhakar V., Niklason L.E. (2003). Decellularized native and engineered arterial scaffolds for transplantation. Cell Transpl..

[33] Uchimura E., Sawa Y., Taketani S., Yamanaka Y., Hara M., Matsuda H., Miyake J. (2003). Novel method of preparing acellular cardiovascular grafts by decellularization with poly(ethylene glycol). J. Biomed. Mater. Res..

[34] Chen R.-N., Ho H.-O., Tsai Y.-T., Sheu M.-T. (2004). Process development of an acellular dermal matrix (ADM) for biomedical applications. Biomaterials.

[35] Hudson T.W., Zawko S., Deister C., Lundy S., Hu C.Y., Lee K., Schmidt C.E. (2004). Optimized Acellular Nerve Graft Is Immunologically Tolerated and Supports Regeneration. Tissue Eng..

[36] Borschel G., Dennis R.G., Kuzon W.M. (2004). Contractile Skeletal Muscle Tissue-Engineered on an Acellular Scaffold. Plast. Reconstr. Surg..

[37] Cartmell J.S., Dunn M.G. (2000). Effect of chemical treatments on tendon cellularity and mechanical properties. J. Biomed. Mater. Res..

[38] Woods T., Gratzer P.F. (2005). Effectiveness of three extraction techniques in the development of a decellularized bone–anterior cruciate ligament–bone graft. Biomaterials.

[39] Badylak S., Lantz G.C., Coffey A., Geddes L.A. (1989). Small intestinal submucosa as a large diameter vascular graft in the dog. J. Surg. Res..

[40] Badylak S., Tullius R., Kokini K., Shelbourne K.D., Klootwyk T., Voytik S.L., Kraine M.R., Simmons C. (1995). The use of xenogeneic small intestinal submucosa as a biomaterial for Achille’s tendon repair in a dog model. J. Biomed. Mater. Res..

[41] Kropp B.P., Eppley B.L., Prevel C., Rippy M., Harruff R., Badylak S., Adams M., Rink R., Keating M. (1995). Experimental assessment of small intestinal submucosa as a bladder wall substitute. Urology.

[42] Gilbert T., Stolz D.B., Biancaniello F., Simmons-Byrd A., Badylak S.F. (2005). Production and characterization of ECM powder: Implications for tissue engineering applications. Biomaterials.

[43] Lin P., Chan W.C., Badylak S.F., Bhatia S.N. (2004). Assessing Porcine Liver-Derived Biomatrix for Hepatic Tissue Engineering. Tissue Eng..

[44] Gilbert T., Sellaro T.L., Badylak S.F. (2006). Decellularization of tissues and organs. Biomaterials.

[45] Järvinen T.A.H., Prince S. (2015). Decorin: A Growth Factor Antagonist for Tumor Growth Inhibition. BioMed Res. Int..

[46] Liang R., Yang G., Kim K.E., D’Amore A., Pickering A.N., Zhang C., Woo S.L.-Y. (2015). Positive effects of an extracellular matrix hydrogel on rat anterior cruciate ligament fibroblast proliferation and collagen mRNA expression. J. Orthop. Transl..

[47] Mewhort H.E., Svystonyuk D.A., Turnbull J.D., Teng G., Belke D.D., Guzzardi D.G., Park D.S., Kang S., Hollenberg M.D., Fedak P.W. (2017). Bioactive Extracellular Matrix Scaffold Promotes Adaptive Cardiac Remodeling and Repair. JACC Basic Transl. Sci..

[48] Xu Q., Shanti R.M., Zhang Q., Cannady S.B., O’Malley B.W., Le A.D. (2017). A Gingiva-Derived Mesenchymal Stem Cell-Laden Porcine Small Intestinal Submucosa Extracellular Matrix Construct Promotes Myomucosal Regeneration of the Tongue. Tissue Eng. Part A.

[49] Keane T., Dziki J., Sobieski E., Smoulder A., Castleton A., Turner N., White L.J., Badylak S.F. (2016). Restoring Mucosal Barrier Function and Modifying Macrophage Phenotype with an Extracellular Matrix Hydrogel: Potential Therapy for Ulcerative Colitis. J. Crohn’s Coliti.

[50] Nezhad Z.M., Poncelet A., De Kerchove L., Gianello P., Fervaille C., El Khoury G. (2016). Small intestinal submucosa extracellular matrix (CorMatrix^®^) in cardiovascular surgery: A systematic review. Interact. Cardiovasc. Thorac. Surg..

[51] Slaughter M.S., Soucy K.G., Matheny R.G., Lewis B.C., Hennick M.F., Choi Y., Monreal G., Sobieski M.A., Giridharan G.A., Koenig S.C. (2014). Development of an Extracellular Matrix Delivery System for Effective Intramyocardial Injection in Ischemic Tissue. ASAIO J..

[52] Soucy K.G., Smith E.F., Monreal G., Rokosh G., Keller B.B., Yuan F., Matheny R.G., Fallon A.M., Lewis B.C., Sherwood L.C. (2015). Feasibility Study of Particulate Extracellular Matrix (P-ECM) and Left Ventricular Assist Device (HVAD) Therapy in Chronic Ischemic Heart Failure Bovine Model. ASAIO J..

[53] Quarti A., Nardone S., Colaneri M., Santoro G., Pozzi M. (2011). Preliminary experience in the use of an extracellular matrix to repair congenital heart diseases. Interact. Cardiovasc. Thorac. Surg..

[54] Witt R.G., Raff G., Van Gundy J., Rodgers-Ohlau M., Si M.-S. (2013). Short-term experience of porcine small intestinal submucosa patches in paediatric cardiovascular surgery. Eur. J. Cardio-Thoracic Surg..

[55] Boyd W.D., Johnson W.E., Sultan P.K., Deering T.F., Matheny R.G. (2010). Pericardial Reconstruction Using an Extracellular Matrix Implant Correlates with Reduced Risk of Postoperative Atrial Fibrillation in Coronary Artery Bypass Surgery Patients. Hear. Surg. Forum.

[56] Yanagawa B., Rao V., Yau T.M., Cusimano R.J. (2013). Initial Experience with Intraventricular Repair Using CorMatrix Extracellular Matrix. Innov. Technol. Tech. Cardiothorac. Vasc. Surg..

[57] Zaidi A.H., Nathan M., Emani S., Baird C., del Nido P.J., Gauvreau K., Harris M., Sanders S.P., Padera R.F. (2014). Preliminary experience with porcine intestinal submucosa (CorMatrix) for valve reconstruction in congenital heart disease: Histologic evaluation of explanted valves. J. Thorac. Cardiovasc. Surg..

[58] Stelly M., Stelly T.C. (2013). Histology of CorMatrix Bioscaffold 5 Years after Pericardial Closure. Ann. Thorac. Surg..

[59] Gerdisch M.W., Shea R.J., Barron M.D. (2014). Clinical experience with CorMatrix extracellular matrix in the surgical treatment of mitral valve disease. J. Thorac. Cardiovasc. Surg..

[60] Biefer H.R.C., Emmert M., Falk V., Sündermann S. (2012). Use of Extracellular Matrix Materials in Patients with Endocarditis. Thorac. Cardiovasc. Surg..

[61] Yanagawa B., Rao V., Yau T.M., Cusimano R.J. (2014). Potential myocardial regeneration with CorMatrix ECM: A case report. J. Thorac. Cardiovasc. Surg..

[62] Drago J., Nurcombe V., Bartlett P.F. (1991). Laminin through its long arm E8 fragment promotes the proliferation and differentiation of murine neuroepithelial cells in vitro. Exp. Cell Res..

[63] Kearns S., Laywell E., Kukekov V., Steindler D. (2003). Extracellular matrix effects on neurosphere cell motility. Exp. Neurol..

[64] Londono R., Badylak S.F. (2014). Biologic Scaffolds for Regenerative Medicine: Mechanisms of In vivo Remodeling. Ann. Biomed. Eng..

[65] Krishnan N.M., Chatterjee A., Rosenkranz K.M., Powell S.G., Nigriny J.F., Vidal D.C. (2014). The cost effectiveness of acellular dermal matrix in expander–implant immediate breast reconstruction. J. Plast. Reconstr. Aesthetic Surg..

[66] Nafisi N., Mahjoub F., Mohseni M.J., Sabetkish S., Khorramirouz R., Tehrani M., Akbari M.E., Kajbafzadeh A.-M. (2017). Application of Human Acellular Breast Dermal Matrix (ABDM) in Implant-Based Breast Reconstruction: An Experimental Study. Aesthetic Plast. Surg..

[67] Tan S.S., Loh W. (2017). The utility of adipose-derived stem cells and stromal vascular fraction for oncologic soft tissue reconstruction: Is it safe? A matter for debate. Surgeon.

[68] Turner A.E., Yu C., Bianco J., Watkins J.F., Flynn L.E. (2012). The performance of decellularized adipose tissue microcarriers as an inductive substrate for human adipose-derived stem cells. Biomaterials.

[69] Majeed A., Borisuk M.J., Padera R.F., Baird C., Sanders S.P. (2016). Histology of Pericardial Tissue Substitutes Used in Congenital Heart Surgery. Pediatr. Dev. Pathol..

[70] Neethling W.M.L., Puls K., Rea A. (2018). Comparison of physical and biological properties of CardioCel^®^ with commonly used bioscaffolds. Interact. Cardiovasc. Thorac. Surg..

[71] Yang F., Murugan R., Ramakrishna S., Wang X., Ma Y.-X., Wang S. (2004). Fabrication of nano-structured porous PLLA scaffold intended for nerve tissue engineering. Biomaterials.

[72] Cao F., Rafie A.H.S., Abilez O.J., Wang H., Blundo J.T., Pruitt B.L., Zarins C., Wu J.C. (2007). In vivo imaging and evaluation of different biomatrices for improvement of stem cell survival. J. Tissue Eng. Regen. Med..

[73] Kurosaka K., Takahashi M., Watanabe N., Kobayashi Y. (2003). Silent Cleanup of Very Early Apoptotic Cells by Macrophages. J. Immunol..

[74] Greene L.A., Tischler A. (1976). Establishment of a noradrenergic clonal line of rat adrenal pheochromocytoma cells which respond to nerve growth factor. Proc. Natl. Acad. Sci. USA.

[75] Bachmann M., Conscience J.-F., Probstmeier R., Carbonetto S., Schachner M. (1995). Recognition molecules myelin-associated glycoprotein and tenascin-C inhibit integrin-mediated adhesion of neural cells to collagen. J. Neurosci. Res..

[76] Ghuman H., Mauney C., Donnelly J., Massensini A., Badylak S.F., Modo M. (2018). Biodegradation of ECM hydrogel promotes endogenous brain tissue restoration in a rat model of stroke. Acta Biomater..

[77] Shen W., Chen X., Hu Y., Yin Z., Zhu T., Hu J., Chen J., Zheng Z., Zhang W., Ran J. (2014). Long-term effects of knitted silk–collagen sponge scaffold on anterior cruciate ligament reconstruction and osteoarthritis prevention. Biomaterials.

[78] Lee H., Woo H.-M., Kang B.-J. (2017). Impact of collagen-alginate composition from microbead morphological properties to microencapsulated canine adipose tissue-derived mesenchymal stem cell activities. J. Biomater. Sci. Polym. Ed..

[79] Arnaoutova I., George J., Kleinman H.K., Benton G. (2012). Basement Membrane Matrix (BME) has Multiple Uses with Stem Cells. Stem Cell Rev. Rep..

[80] Anlar B., Atilla P., Çakar A.N., Kose M.F., Beksaç M.S., Dagdeviren A., Akçören Z. (2002). Expression of adhesion and extracellular matrix molecules in the developing human brain. J. Child. Neurol..

[81] Flanagan L.A., Rebaza L.M., Derzic S., Schwartz P.H., Monuki E.S. (2006). Regulation of human neural precursor cells by laminin and integrins. J. Neurosci. Res..

[82] Marastoni S., Ligresti G., Lorenzon E., Colombatti A., Mongiat M. (2008). Extracellular Matrix: A Matter of Life and Death. Connect. Tissue Res..

