# Correction: Gębczak et al. Evaluation of PC12 Cells’ Proliferation, Adhesion and Migration with the Use of an Extracellular Matrix (CorMatrix) for Application in Neural Tissue Engineering. *Materials* 2021, *14*, 3858

**DOI:** 10.3390/ma15207217

**Published:** 2022-10-17

**Authors:** Katarzyna Gębczak, Benita Wiatrak, Wojciech Fortuna

**Affiliations:** 1 Department of Basic Medical Sciences, Wroclaw Medical University, Borowska 211, 50-556 Wroclaw, Poland; 2 Department of Pharmacology, Faculty of Medicine, Wroclaw Medical University, Mikulicza-Radeckiego 2, 50-345 Wroclaw, Poland; 3 Department of Neurosurgery, Wroclaw Medical University, Borowska 213, 50-556 Wroclaw, Poland

## Missing Funding

In the original publication [1], the funder **National Science Centre, Poland**, **grant no. DEC-2012/06/M/NZ4/00138** granted to **Wojciech Fortuna** was not included. The authors state that the scientific conclusions are unaffected. This correction was approved by the Academic Editor. The original publication has also been updated.
